# Marked Effects of Larval Salt Exposure on the Life History and Gut Microbiota of the Malaria Vector *Anopheles merus* (Diptera: Culicidae)

**DOI:** 10.3390/insects13121165

**Published:** 2022-12-16

**Authors:** Ashmika Singh, Nashrin F. Patel, Mushal Allam, Wai-Yin Chan, Thabo Mohale, Arshad Ismail, Shüné V. Oliver

**Affiliations:** 1 Centre for Emerging Zoonotic and Parasitic Diseases, National Institute for Communicable Diseases of the National Health Laboratory Service, Johannesburg 2192, South Africa; 2 Wits Research Institute for Malaria, School of Pathology, Faculty of Health Sciences, University of the Witwatersrand, Johannesburg 2193, South Africa; 3 Department of Genetics and Genomics, College of Medicine and Health Sciences, United Arab Emirates University, Abu Dhabi 15551, United Arab Emirates; 4 Sequencing Core Facility, National Institute for Communicable Diseases of the National Health Laboratory Service, Johannesburg 2131, South Africa; 5 Department of Biochemistry, Genetics and Microbiology, Forestry and Agricultural Biotechnology Institute, University of Pretoria, Pretoria 0028, South Africa; 6 Department of Biochemistry and Microbiology, Faculty of Science, Engineering and Agriculture, University of Venda, Thohoyandou 0950, South Africa

**Keywords:** osmoregulation, development, diversity, bacterial abundance

## Abstract

**Simple Summary:**

*Anopheles merus* is a malaria-transmitting mosquito with the unusual ability to breed in a range of saltwater concentrations. Although previous studies have looked at the consequences of this capacity on the immature mosquitoes present in the saltwater, what this means for the adults that emerge from this water is unknown. In this study, we look at the consequences of breeding in a range of saltwater concentrations on laboratory-bred *An. merus*. We found that high salt concentrations delayed the time to pupation but does not affect adult longevity. Therefore, although the larvae are at risk for a longer period, it does not affect the lifespan of the adult. Increased salt exposure also results in increased insecticide tolerance, which makes them harder to control. Finally, the salt concentration affects the composition of the mosquito’s gut microbiota. The significance of this is that the gut bacteria composition affects the capacity to transmit the malaria parasite. This study suggests that due to changes in the gut bacteria, mosquitoes that breed in very salty water are less likely to carry the malaria parasite in their gut. Therefore, the saltiness of the water that these mosquitoes breed in may affect how well they transmit malaria.

**Abstract:**

*Anopheles merus* can breed in a range of saltwater concentrations. The consequences of this ability on the life history of adult *An. merus* are poorly understood. This study examined the effects of exposure to 0, 2.1875, 4.375, 8.75, and 17.5 g/L of sodium chloride on *An. merus*. The effects on larval development, adult longevity, fertility, and fecundity, as well as deltamethrin tolerance were examined. The effect of larval salt exposure on the expression of *defensin-1* in adults was examined by quantitative Real-Time PCR. Finally, the effect of the larval salt concentration on microbial dynamics was assessed by 16S Next Generation Sequencing. High concentrations of saltwater increased larval development time and number of eggs laid, as well as deltamethrin tolerance. Larval exposure to salt also reduced the expression of *defensin-1*. The exposure also had a significant effect on microbial diversity in larvae and adults. The diversity of larvae decreased once adults emerged. Salt-tolerant bacterial genera predominated in larvae but were absent in adults. High salt concentrations resulted in greater abundance of *Plasmodium*-protective genera in adults. Although this study was conducted on a laboratory strain of *An. merus*, these data suggest that osmoregulation has a significant effect on the life history of the species with potential epidemiological consequences.

## 1. Introduction

Insects are one of the most prolific organisms in terrestrial and fresh-water environments. By contrast, their success in marine environments is more limited. As such, to the best of our knowledge, the hemipteran genus *Halobates* is the only genus of insects that are strictly marine insects. Other insects that are considered marine insects are associated with coastal habitats such as estuaries and saltmarshes, rather than relying solely on the ocean [1]. This pattern is also true for many species of mosquitoes, where the larval stage of the mosquito occurs in fresh water 95% of the time, owing to the fact that majority of mosquito species cannot tolerate osmotic stress that exceeds the osmolarity of the haemolymph. This critical concentration is approximately 300 osmol/L, which corresponds to roughly 30% seawater [2]. However, approximately 5% of mosquito species are more osmotolerant and are capable of surviving hypersaline conditions [3].

The most well-known osmotolerant mosquitoes belong to the genus *Aedes*. This includes the saltmarsh mosquito *Aedes detritus.* The New Zealand salt pool mosquito *Opifex fuscus* is another example of a marine mosquito [4]. Furthermore, while *Aedes* mosquitoes are generally considered obligate freshwater mosquitoes [5], there have been reports of salt-tolerance in *Aedes detritus* [6]. Salt tolerance has also been reported in other significant vector species, where *Aedes albopictus* was found to be the most salt tolerant, followed by *Anopheles coluzzii*, *Aedes aegypti*, *Culex quinquefasciatus*, and *Anopheles gambiae*, with *Culex. pipiens* being the least salt tolerant [5]. Another study examined the long-term effects of salt exposure on the competition between *Ae. aegypti* and *Ae. albopictus*. This study found that *Ae. aegypti* was more tolerant to long-term exposure, but that salinity did not affect the competition between the two species. The preference for saline larval breeding conditions may be due to reduced competition and predation in such conditions. Furthermore, such breeding conditions are high in nutrients, providing a competitive advantage to mosquitoes that can inhabit these niches [4].

Apart from *An. coluzzi*, other salt-tolerant *Anopheles* mosquitoes include *Anopheles aquasalis* [7], *Anopheles sundaicus* [8], and *Anopheles multicolor* [9]. Furthermore, within the *An. gambiae* complex, *Anopheles bwambae*, *Anopheles melas*, and *Anopheles merus* are salt-tolerant species [10]. It is also notable that *Anopheles arabiensis* in Mauritania showed variable saline tolerance, with a 75% survival in 17.5 g/L saltwater [9]. The salt tolerance of the *An. gambiae* complex has significant implications for vector control in Africa because while *Anopheles bwambae* is limited in distribution, *An. melas* and *An. merus* have wider distribution. *Anopheles melas* is found on the west coast of Africa, while *An. merus* is found on the east coast of Africa and is found as far south as South Africa [10]. Thus, *An. merus* is a secondary dominant vector species in Africa and is commonly found in southern Africa where it has been found to be a vector of bancroftian filariasis in Tanzania [11]. *An. merus* also tends to dominate in the dry season [11]. In South Africa, this species is found in all three malaria-endemic provinces and is the most prevalent member of the *An. gambiae* complex in the Mpumalanga province [12]. Although *An. merus* has been implicated in malaria transmission in Tanzania and Madagascar, it has not been implicated in malaria transmission in South Africa yet [12]. Despite this, there is a possibility that the species could contribute to the low-level outdoor transmission of malaria, which is known as residual malaria [13].

The osmotolerance in *An. merus* is an unusual characteristic and is thus poorly examined. The effect of salt dosage on *An. merus* development has previously been described [14]. The transcriptomic response to osmotic stress in euryhaline (salt tolerant) and stenohaline (freshwater) malaria-vector sibling species has also been described with], the transcriptome of larvae reared in salt water were investigated [15]. However, while the effect of breeding in variable salt concentrations on mosquito larvae has been examined a few times, the effect on the emerging adults has not been examined. This is true for *An. merus* as well. Therefore, although it has been demonstrated that immune genes are largely downregulated in larvae with increasing salt concentration [14], it is not known whether this has a carry-through effect to the adult. Therefore, in the current study, the effect of variable larval salt concentrations on the antimicrobial peptide defensin-1 was examined. Like most defensins, the antimicrobial peptide defensin-1 is a cysteine-rich peptide, with activity against Gram-positive bacteria and fungi [16]. Defensin-1 is also expressed in the gut [17]. This peptide was chosen as it has been associated with saline tolerance in plants [18]. The paucity of information on this topic is problematic, as it is well known that the larval environment dictates adult fitness, because larval environment affects factors such as adult longevity, size, fertility, and fecundity [19,20,21,22]. Yet, the effect of osmoregulation on mosquito life history and malaria transmission is poorly understood. This is particularly true for solutes in the water ranging from innocuous pollutants such as organic fertiliser [23], to toxic pollutants such as herbicides and heavy metals that may contribute to the need for mosquito species to be adapted to higher salt concentrations in breeding waters [24,25].

Another factor that is not often considered is the effect of the larval environment on the adult gut microbiota. The gut microbiota of mosquitoes is essential for development and pupation [26,27] and plays a critical role in adult life history [28,29]. Crucially, gut microbiota plays an important role in the capacity to transmit the *Plasmodium* parasite in *Anopheles* mosquitoes [30,31]. There has been a range of studies that have demonstrated direct effects on both the bacteria on the parasite [32]. There has also been demonstration of the indirect effects of gut bacteria on *Plasmodium* through modulation of the immune system [33]. Gut microbiota are therefore a potential target for vector control interventions [34,35], and as such, understanding the composition of the gut microbiome is an important consideration for vector biologists. As the adult gut microbiome is impacted by the larval environment [36,37,38], it is possible that variable salt concentrations could differentially affect the gut microbiota of salt-tolerant species. This is likely, therefore, to have epidemiological consequences.

This study therefore aims to address this lack of information of the effects of breeding in variable salt concentrations on adult *An. merus*. This will be accomplished by examining the effect of larval salt concentration on larval development, adult longevity, fertility and fecundity, insecticide tolerance, and gut microbiota.

The *An. merus* strain, MAFUS, used in this study is routinely reared in 17.5 g or 50% seawater. Although there are reports of South African *An. merus* breeding in water as salty as 123% seawater, this laboratory strain was not capable of surviving in higher salt concentrations. Therefore, although there may be wild *An. merus* in South Africa capable of surviving higher salt concentrations, this concentration was high enough to exclude other members of the *An. gambiae* complex [14], as well as being the highest concentration that the strain could tolerate (unpublished data). Therefore the 17.5 g (50% seawater) represented the highest salt concentration used, and all concentrations were based on dilutions of this value. As such, the concentrations used in this study are substantially higher than a previous study on the effects of salt on *Ae. albopictus* larvae [39] and are similar to the previous study of the effects of salt concentration on *An. gambiae* larvae [14]. It is hypothesised that variable salt concentrations will differentially affect a range of life history traits and expression of the antimicrobial peptide defensin-1, as well as the composition of the gut microbiome. As the strain is routinely reared in 17.5 g salt, it is hypothesised that the most marked effects will be in adults emerging from larvae reared in this concentration.

## 2. Materials and Methods

### 2.1. Strain and Larval Treatment

MAFUS is an *An. merus* strain colonised from Mafayeni, Kruger National Park, Limpopo, South Africa in 2012. The strain has low-level tolerance to insecticides. In the colonisation period, the strain was reared in 17.5 g/L of sodium chloride. This concentration represents the 50% seawater concentration of sea water. As such 8.75 g/L represented the 25% seawater concentration, 4.375 g/L represented the 12.5% seawater concentration, 2.1875 g/L represented the 6.25% seawater concentration, and the water without salt added constituted the 0% salt concentration.

For each experiment, 200 first-instar larvae (less than 24 h old) were collected. These were transferred to 1000 mL of each concentration of water in a plastic container (214 × 155 × 80 mm). The larvae were fed an equal amount of larval food until pupation and were reared under the conditions and diet described in [40]. In brief, mosquitoes were reared at 25 °C (±2 °C) and 80% humidity (±5%). The mosquitoes were subjected to a 12:12 h light-dark cycle with a 30 min dusk/dawn cycle. Adults were maintained on a 10% sucrose solution.

### 2.2. The Effects of Variable Salt Concentration on Life History

#### 2.2.1. Development Time

Fifty first-instar larvae were set up as described earlier. Development time was measured as the time to pupation. This experiment was replicated three times using cohorts originating from three separate egg batches. The development time was assessed using a Kaplan–Meier [41] estimator with a log-rank test [42] as the measure of significance.

#### 2.2.2. Adult Longevity

From each concentration, 25 males and 25 females were kept in a cage with ad libitum access to 10% sucrose. The sucrose solution was changed twice a week. The adults were collected upon emergence and placed in a cage. Throughout the duration of the experiment, the females were not allowed access to blood. Mortality was recorded daily, and cadavers were removed once a day. The experiment was considered complete once all the individuals were dead. This experiment was replicated three times. Longevity was analysed using a Kaplan–Meier [41] estimator with a log-rank test [42] as the measure of significance.

#### 2.2.3. Fertility and Fecundity

From each concentration, 50 males and 25 females were collected and allowed a blood meal at the ages of 3 and 7 days. At 11 days, the cage was allowed to oviposit for 18 h. The egg plate was removed, and the eggs were allowed to hatch for 72 h. The number of larvae and remaining unhatched eggs were counted. This experiment was replicated four times using adults that were set up from independent larval preparations. Bloodmeals were provided by a single consenting human volunteer (SVO) as per the ethics waiver S-Oliver 03-01-2018-O from the University of the Witwatersrand. Data were analysed using a Chi-squared test.

#### 2.2.4. Insecticide Tolerance

At the age of 3 days, non-blood-fed females were used to determine lethal time using a CDC bottle bioassay. The adults were exposed to 0.001 mg/mL of deltamethrin for either 2, 4, 8, 16, or 32 min as per [43]. Adults were removed from the bottles after the set time and were allowed ad libitum access to sucrose. Mortality was scored 24 h after exposure. Lethal times were calculated by probit analysis [44]. Controls consisted of the unexposed mosquitoes (0 min—environmental control) and mosquitoes exposed to a bottle coated in acetone only (solvent control). The experiment was replicated five times. Data were analysed for normality using the Shapiro–Wilk test and the means were compared using a One-Way Analysis of Variance (ANOVA) with an alpha value of 5% [45]. A Tukey test was used as a post-hoc test [46].

### 2.3. The Effect of Larval Salt Concentration on the Expression of Defensin-1

#### 2.3.1. RNA Extraction and Quality Control

RNA was extracted from females from each of the different concentrations. Five replicates of 3 non-blood-fed 3-day-old females were used in the extraction. RNA was extracted using a column-based Quick-RNA™ MiniPrep kit (Zymo Research: R1054). Samples were mechanically homogenised using a Gene^®^ 2 vortex according to manufacturer’s instructions. Purity and concentration of the extracted RNA were assessed using a Nanodrop™ 2000 C and integrity was confirmed by TapeStation™ 4200 system analysis with RNA ScreenTape^®^ (Agilent Technologies, Santa Clara, CA, USA). RNA integrity was also assessed using 1% 0.5× Tris-Borate-EDTA (TBE) agarose gel electrophoresis at 70 V, 90 mA for 30 min. Samples were sized using a RiboRuler™ High Range RNA Ladder (Thermo Scientific™: SM1821).

#### 2.3.2. Analysis of Transcript Levels

The extracted RNA was used to generate 1000 nM of complementary DNA (cDNA) in replicates of three, using the iScript™ cDNA Synthesis Kit (Bio-Rad, Rosebank, South Africa: 1708891) according to manufacturer’s instructions. The cDNA (100 nM) was used as a template for the quantitative Real-Time Polymerase Chain reaction (qRT-PCR) reaction in SsoAdvanced™ Universal SYBR^®^ Green Supermix (Bio-Rad: 1725270) according to the manufacturer’s instructions with the following cycling conditions: DNA denaturation at 98 °C (3 min) followed by 40 cycles at 95 °C (10 s) with a final extension at 60 °C (30 s). The sequences of the primers and reference genes used in this study are listed in Supplementary Table S3. Differences in transcripts levels were assessed by the Pfaffl method [47] by One-Way ANOVA using Bio-Rad CFX maestro™ software (version 4.0.2325.0418: 2017)

### 2.4. The Effect of Larval Exposure to Variable Salt Concentration on An. merus Gut Microbiota

#### 2.4.1. Midgut Resection and DNA Extraction

Larval activity was minimised by chilling at 4 °C. The adult female mosquitoes were cold killed at −70 °C before their midguts were resected. The dissection procedure took place under sterile conditions. All equipment as well as the mosquitoes were sterilised with 70% ethanol and 3% hydrogen peroxide. The midguts from the respective samples were dissected using an Olympus SZ2-ILST stereomicroscope at a magnification of 40×. Five replicates of 3 midguts each in 200 µL of 0.1 M Phosphate Buffered Saline (PBS) at pH 7.2 were prepared.

The DNeasy Blood & Tissue Kit^®^ (Qiagen: 69506) was used to extract total genomic DNA from the midgut of the mosquito. The midguts were centrifuged at 5000× *g* and approximately 150 µL of PBS was discarded; the remaining PBS and midguts were briefly vortexed and added to new tubes. The protocol was then followed as per manufacturer’s instructions.

#### 2.4.2. Amplification of the V3–V4 Hypervariable Region of the 16S rRNA Gene

Universal bacterial primers were used to amplify the V3–V4 hypervariable region of the bacterial 16S rRNA gene. The primers used were 4 nmol Ultramer^®^ DNA Oligo 55 bases (Integrated DNA Technologies, Coralville, IA, USA) [48]. Primer sequences are detailed in Supplementary Table S1. Library preparation was performed according to the standard instructions of the 16S Metagenomic Sequencing Library Preparation protocol (IlluminaTM, Inc., San Diego, CA, USA). The Qubit^®^ High Sensitivity dsDNA Assay Kit (Thermo Fisher Scientific: Waltham, MA, USA) was used to quantify indexed amplicons. The amplicons were then visualised and sized using the 4200 TapeStation™ (Agilent Technologies: Santa Clara, CA, USA).

Normalized libraries were pooled and denatured using 0.2N NaOH. Libraries were subsequently sequenced on the MiSeq platform using the MiSeq Reagent kit v3 (Illumina) and paired-end 2 × 300 bp sequencing was performed at the Sequencing Core Facility, National Institute for Communicable Diseases, South Africa.

#### 2.4.3. Bioinformatic Analysis

Several quality control steps were taken to ensure clean data were used for clustering. Sequences were filtered and trimmed. Raw reads were trimmed with a 0.05 quality limit. The SILVA 16S v132 database was used to cluster data at a 99% similarity threshold. The OTUs were assigned to sequences based on 97% identity. The OTU table was then used to prepare a bar plot to visually represent the midgut bacteria of each respective sample.

Raw reads were used for bacterial diversity analysis. The raw reads were quality-controlled and filtered (Q > 20 and length > 50 bp) using the programmes fastqc (v0.11.8) and Trim Galore (v0.6.4_dev; https://github.com/FelixKrueger/TrimGalore, accessed on 18 May 2022), respectively [49,50]. Trim Galore was also used for adapter removal.

All the downstream analyses were performed in R (v3.6.1). This included classification, abundance estimations, statistical analysis, and visualization. The dada2 package (v1.12.1) [51] was used to pre-process clean reads. This included quality inspection, trimming, dereplication, merging paired-end reads, and removal of chimeric sequences.

The SILVA reference database (v138; https://zenodo.org/record/1172783#.XvCmtkUzY2w: accessed on 18 May 2022) was used to assign taxonomy to the amplicon sequence variants (ASVs). The ASV abundance estimates were determined using training sequence sets based on the SILVA database.

The phyloseq package (v1.28.0) [52], ggplot2 (v3.2.1), and AmpVis2 package (v2.6.4) [53] were used to determine ordinations for beta diversity, abundance bar plots, alpha diversity, and richness estimates, and heatmaps. Data generated using clustering in Non-metric Multidimensional Scaling (NMDS) were assessed using PERMANOVA (permutation test with pseudo-F ratios) as implemented in the adonis function in the vegan package (https://github.com/vegandevs/vegan, accessed on 18 May 2022).

Alpha diversity between groups was compared using Kruskal–Wallis rank-sum tests. Overlapping species diagrams were generated using Venn Diagram (v1.6.20) and UpsetR (v1.4.0) [54]. DESeq2 (v1.24.0) [55] was used to determine differential abundance analysis between sample groups.

## 3. Results

### 3.1. The Effect of Larval Salt Concentration on Life History

#### 3.1.1. Development Time

**Figure 1:**
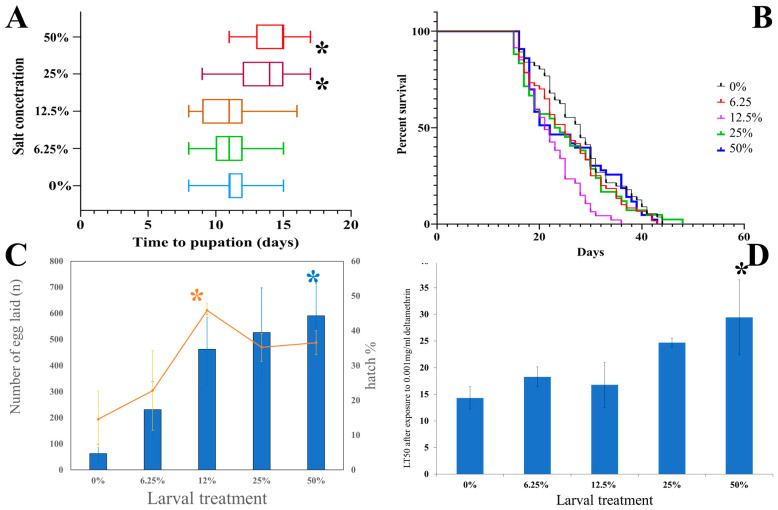
The effect of variable larval salt concentration on life history traits of Anopheles merus. (**A**) Time-to-pupation in different larval salt concentrations. Asterisks (*) indicate a significant difference from the untreated control. (**B**) Adult longevity of female *An. merus* after rearing in different larval salt exposures. (**C**) Fertility and fecundity of adults reared in different larval salt concentrations. Blue bars represent number of eggs, and the orange line represents hatch percentage. Blue asterisks represent a significantly different number of eggs from the control. Orange asterisks represent a significantly different hatch percentage from the untreated control. (**D**) Deltamethrin lethal time of adults reared in different larval salt concentrations. Asterisks indicate a significant difference from the untreated control.

Larvae that developed in the 50% (17.5 g/L) and 25% (8.75 g/L) salt concentration conditions took significantly longer to pupate than the other treatments (log-rank test: *p* < 0.01, χ^2^ = 112.15, df = 2). There was no significant difference in development time of the larvae between the 0%, 6.25% (2.188 g/L), and 12.5 % (4.375 g/L) salt concentrations (*p* = 0.85, χ^2^ = 0.31, df = 2) (Figure 1A).

#### 3.1.2. Adult Longevity

There was no significant difference in the male adult longevity regardless of treatment (log-rank test: *p* = 0.22, χ^2^ = 5.79 df = 4). For females, there was a significant effect on longevity (*p* < 0.01, χ^2^ = 16.81 df = 4), but the effect was only due to the reduced longevity of the 25% treatment (Figure 1B).

#### 3.1.3. Fertility and Fecundity

Larval salt exposure affected adult fertility and fecundity. There was a significant difference in fecundity (number of eggs laid) (Chi-squared: *p* < 0.01; χ^2^ = 2767 df = 12). A Tukey post-hoc test showed that the significance was due to the difference between the 0% and 50% treatments. There was also a significant difference in fertility (percentage of eggs hatched) (*p* = 0.03 χ^2^ = 124.8 df = 12) (Figure 1C).

#### 3.1.4. Insecticide Tolerance

Larval salt treatment altered sensitivity to deltamethrin when comparing LT50 values (one-way ANOVA: *p* = 0.03, F_(4, 21)_ = 3.20). A Tukey post-hoc test demonstrated that this was due to the differences between 0% and 50% treatments, without any difference between the 6.25, 12.5, and 25% treatments (Figure 1D). This was emphasised by the LT99 values, where the 50% treatment had the highest lethal time (*p* < 0.01, F_(4, 21)_ = 6.74).

### 3.2. The Effect of the Larval Salt Concentration on Defensin-1 Expression

**Figure 2:**
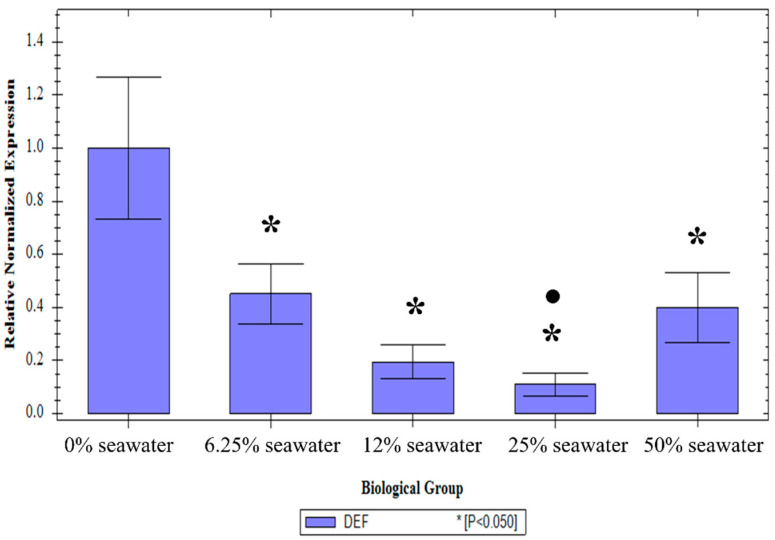
Relative normalised expression of defensin-1 transcripts from *An*. *Merus* adults reared in different larval salt concentrations. Asterisks (*) denote a significant difference from the untreated control. Circles (●) indicate a significantly lower expression in the treated groups.

There was no effect of the treatment on the expression of the 18S transcript (one-way ANOVA: *p* = 0.73, F_(4, 40)_ = 0.50) or the 26S transcript (*p* = 0.97, F_(4, 40)_ = 0.13). The treatment, however, did result in a significant change in defensin-1 transcript level compared to the 0% treatment (*p* < 0.01, F_(4, 40)_ = 21.31). A Tukey post-hoc test showed that all the treatments had significantly lower defensin-1 transcript levels than the 0% treatment. Furthermore, the 6.25% treatment had a significantly higher transcript level than the 12.5% treatment (Holm–Bonferroni corrected *p*-value: *p* < 0.01) and the 50% treatment (corrected *p*-value: *p* < 0.01). The 50% treatment also had a significantly higher transcript level than the 25% treatment (corrected *p*-value: *p* = 0.03), but not the 6.25% or 12.5% treatment (corrected *p*-values: *p* = 0.52 and *p* = 0.23 respectively) (Figure 2).

### 3.3. The Effect of Larval Salt Concentration on An. merus Microbiota

Bar plots summarising the species found in the Next Generation Sequencing study, as well as the rarefaction, plots can be found in Supplementary Figures S1–S4, while the OTU tables can be found in Supplementary Tables S1–S4.

#### 3.3.1. Alpha and Beta Diversity

Alpha diversity is a measure of diversity within the sample. As such, α-diversity is a measure of the microbial diversity within each treatment. Alpha diversity can be described in terms of species richness, which is a measure of the number of species present, or the absolute abundance. This is described by the ACE and Chao1 index. It can also be described in terms of species evenness, or relative abundance within a community. This can be described by the Shannon and Simpson indices. This analysis gives an indication of the direct effect of the treatment on the local microbiota.

**Figure 3:**
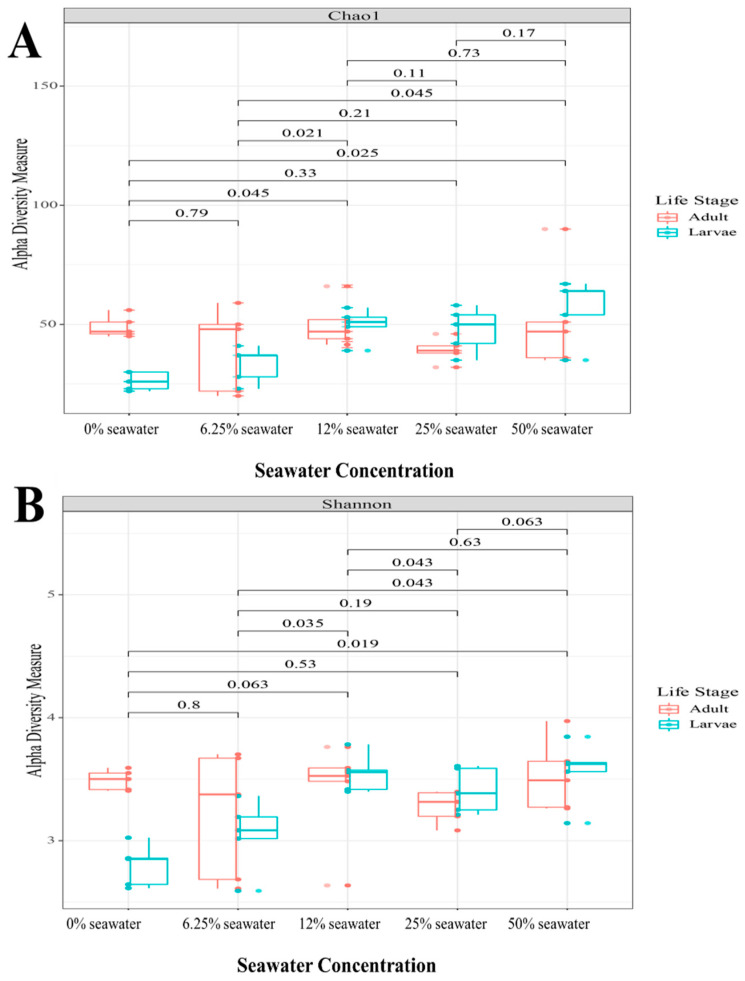
Alpha diversity of adult *An. merus* gut microbiota. (**A**) Chao1 index (species richness). (**B**) Simpson index (species evenness). Values indicate *p*-values from Kruskall–Wallis ANOVA.

When examining α-diversity, there was a marked difference in the effect of larval salt exposure on species richness and evenness. There was no significant difference in species richness, as assessed by the Chao1 index (Figure 3A) and ACE index. By contrast, there were significant differences in species evenness. When examining the Shannon index, there were significant differences between the 0% and 50% treatments (Kruskal–Wallis ANOVA: *p* = 0.02), the 6.25% and 12.5% treatments (*p* = 0.04), the 6.25% and 50% treatments (*p* = 0.04), and the 12.5% and 25% treatments (*p* = 0.04) (Figure 3B). A similar pattern was observed for the Simpson index. There was a significant difference between 0% and 50% (*p* = 0.03), and 6.25% and 50% (*p* = 0.04).

Beta diversity refers to the change in species between communities. For microbiome studies, β-diversity involves a quantification of the similarity and dissimilarity between communities. In this study, β-diversity was measured by the Bray–Curtis index and the ordination method used was the non-metric multi-dimensional scaling (NMDS) method. This analysis allows a comparison of the effect of different larval salt exposures on microbiota.

**Figure 4:**
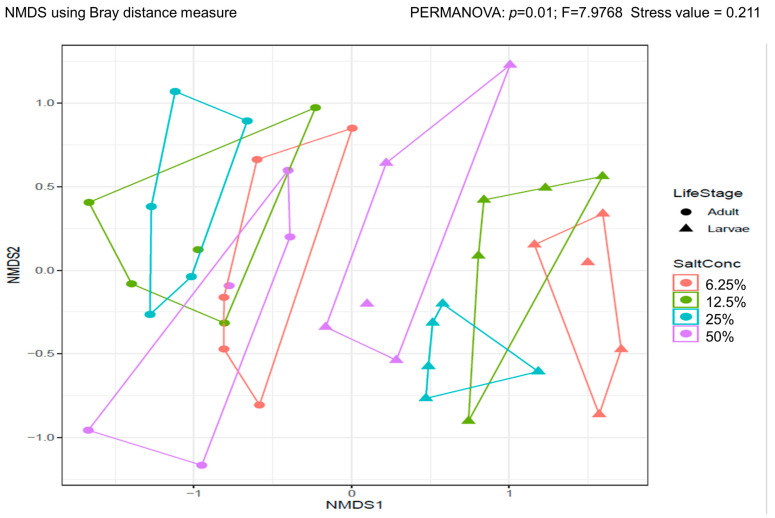
Beta diversity of 4th instar larvae and adult *An. merus* reared in differing larval salt concentration. Non-Metric Multidimensional Scaling plot of β-diversity. Larvae (triangles) from different salt concentrations cluster separately from the adults (circles) and are diverse from each other. Adults are also less diverse and their diversities overlap.

There was a significant difference in the β-diversity of the larvae and the adults (PERMANOVA: *p* = 0.01, F = 7.98, stress value = 0.21). While the β-diversity of the larvae differed from each other, the diversity of the adults overlapped (Figure 4).

#### 3.3.2. Overlapping Species

**Figure 5:**
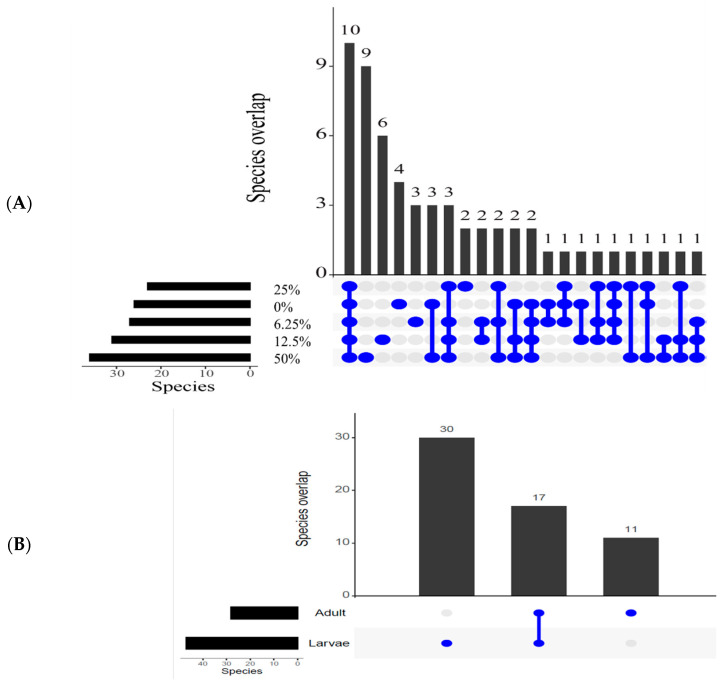
Overlapping gut microbiota in *An. merus* reared in varying larval salt concentrations. (**A**) Overlapping species in adults originating from larval different treatments. (**B**) Overlapping species in adults and larvae. Individual dots indicate numbers of individual species. Joined dots indicate numbers of shared species between the relevant concentrations.

In order to understand how many species the treatments had in common, the number of overlapping species was analysed. All treatments had eight families in common, as well as 10 genera and 10 species. The 50% treatment had the most unique families, genera, and species (six, nine, and nine respectively). By contrast, the 25% treatment was the least unique, with only a single unique family and no unique genera or species. This was followed by the 0% treatment, which had only four unique genera and species, and only two unique families (Figure 5).

When looking at life stages, larvae were always more diverse. This was true for unique families (17:5, with 13 shared), genera, and species (30:11, with 17 shared).

#### 3.3.3. Relative Abundance

**Figure 6:**
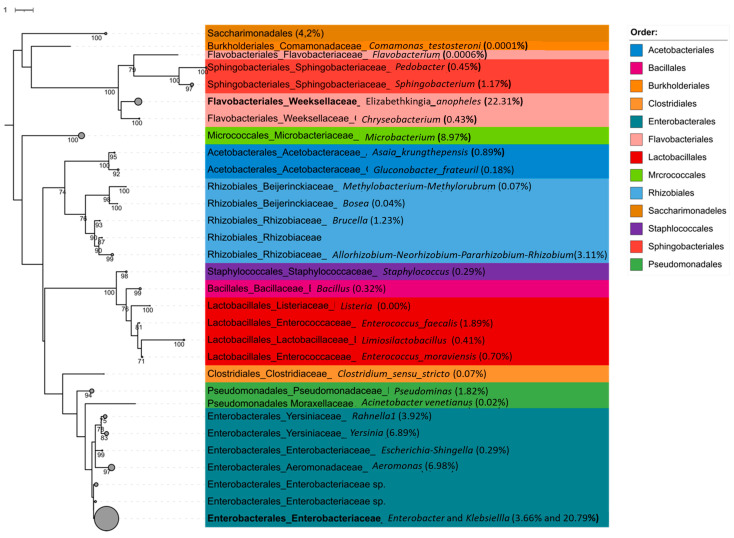
Phylogenetic tree of 16S rRNA sequences from *Anopheles merus* adults and larvae from different salt concentrations. Using the alignment of classified OTU sequences, the maximum likelihood tree was constructed using RAxML with the GTRGAMMAIX model and 1000 bootstraps. Taxa under the same genus were collapsed and their overall OTU abundance percentages are provided within the parentheses.

In order to understand which species are relevant in each treatment, it is important to understand which species predominate in each treatment. This can be achieved by calculating the relative abundance of species in each treatment. In this analysis, the species abundant in each treatment were compared (either positively or negatively) to the 50% seawater treatment, as this is the concentration that the strain is bred in routinely. The species found are summarised in Figure 6.

The phyla most represented in larvae are Acinetobacteriota, Patescibacteria, Firmicutes, Bacteroidota, and (the most dominant) Proteobacteria. The 50% treatment was used as a baseline, as this is the standard concentration the strain is reared in. For the larvae, the 50% treatment generally had more genera differentially expressed. This was true when compared to the 0% (12 genera vs. 18), 6.25% (10 genera vs. 15), and 25% treatments (12 genera vs. 14). The exception was the 12.5% treatment, in which the treatment had 16 genera represented, compared to 13 in the baseline (See Supplementary Figure S4).

**Figure 7:**
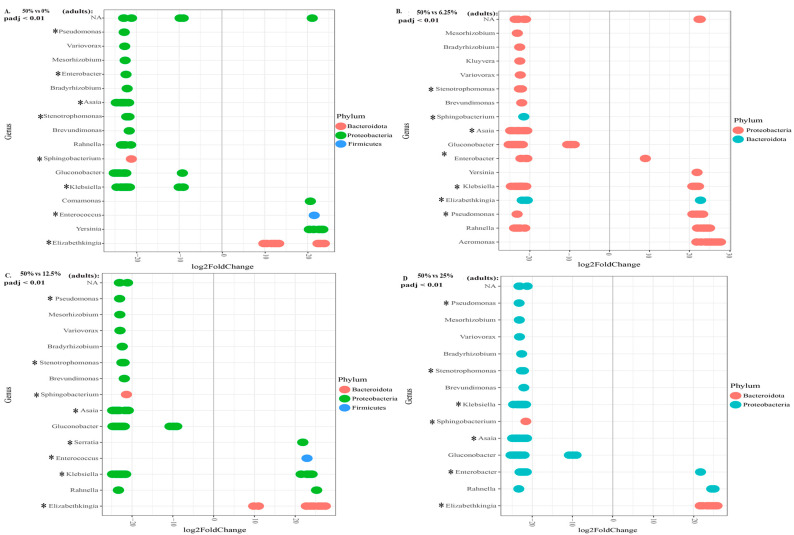
Differential abundance of microbiota in treatments compared to a 50% seawater treatment. Genera to the left of 0 are abundant in the 50% treatment, while genera to the right of 0 are abundant in the comparative concentration. (**A**) Adult 50% compared to 0% treatment. (**B**) Adult 50% compared to 6.25% treatment. (**C**) Adult 50% compared to 12.5% treatment. (**D**) Adult 50% compared to 25% treatment. Genera with asterisks have been associated with protection against *Plasmodium*.

This is even more marked in adults. When comparing the 50% treatment to the 0% treatment, the former had twelve genera more abundantly represented, compared to the four in the 0% group (Figure 7A). For the 50% and 6.25% treatments, there were fourteen genera compared to seven, respectively (Figure 7B). For the 50% and 12.5% treatments, there were eleven genera compared to five, respectively (Figure 7C). Finally, for the 50% compared to 25% treatment, there were twelve genera compared to three (Figure 7D). All the genera considered abundantly represented were significant an alpha value of 0.01.

## 4. Discussion

Despite *An. merus’* status as a secondary vector species, there is remarkably little information on this species. It is worth noting that this study is based on a laboratory strain of *An. merus*, which is not a commonly colonised strain. In White et al. [14], one of the few comprehensive studies on salinity tolerance in the species, the MAF strain was used. This strain (MRA-1156) originated in the same region as the MAFUS strain but is routinely reared in 17.56 g/L rather than 15.85 g/L. This suggests that the findings in MAFUS may be similar to that of MAF.

The two higher concentrations of salt resulted in slower time-to-pupation. This reduced rate of development has been seen when *An. arabiensis* was exposed to metal salts [24]. Although this can be attributed to the toxicity of the heavy metal salts, high salt concentrations are toxic to *An. merus* as well. As *An. merus* coped better with salt exposure under chronic rather than acute exposure, this suggests that osmotic stress is a challenge that this euryhaline species needs to adapt to [14]. However, the lack of effects on longevity suggests that although challenging, exposure to high salt concentrations is not as toxic as exposure to metals or herbicides [24,25], but also not as much of an advantage as exposure to fertiliser, which increased longevity [23,43]. Although, to the best of the authors’ knowledge, there is no record of a similar study in mosquitoes, there is a striking similarity in the response to sodium chloride by the Fall armyworm, *Spodoptera frugiperda*. Namely, the salt exposure increased larval development but did not affect adult viability or reproductive capacity in this species either [56].

There are very few studies on the effects of larval sodium chloride exposure on mosquitoes. Typically, the research has tended to be focussed on *Aedes* species, as this genus has more saline-tolerant species. Furthermore, where saline effects were examined in *Anopheles* mosquitoes, usually *An. merus*, it tended to be focussed on the effects on the larvae. This study, therefore, adds to a very small body examining the effect of larval salt concentration on adult life history. A study of the effect of salt concentrations on Aedes albopictus found that oviposition decreased with increasing salt concentration [39]. This was not examined in this study, as all larvae were allowed to oviposit in fresh water, which the aforementioned study suggests would have resulted in the greatest number of eggs produced. In the Guo et al. [39] study, wild *Ae. albopictus* demonstrated a marked pattern of reduced hatching beyond 3% salt concentration, while hatching from 0 to 2% salt was relatively consistent. Although the concentrations tolerated by this An. merus strain were substantially higher, the hatch percentage peaked at 25% salt and decreased with higher concentrations. The increased number of eggs laid by the offspring exposed to higher concentrations of salt is a reflection of the laboratory strain being selected for high saltwater tolerance. This is therefore unlikely to be reflective of patterns in wild *An. merus* breeding in fresh water.

The effect of high salt concentration on insecticide tolerance is not unprecedented. Various larval exposures to stressors in the environment have increased insecticide tolerance [23,43]. A previous study also indicated the upregulation of various cytochrome P450 genes in *An. merus* in response to osmotic stress [15]. This is congruent with an increased tolerance to pyrethroids [57,58,59,60]. What is notable is that this effect is dose dependent, and that a high salt concentration is needed to alter insecticide tolerance in the strain. Therefore, this is likely only to be of epidemiological concern under exceptional circumstances.

In plants, defensins play a role in defence against osmotic stress [18]. This was the motivation for examining the effect of larval salt exposure on the expression of this antimicrobial peptide. There was a marked effect of the salt exposure on defensin expression, with all treatments resulting in a significant decrease in expression. This is not unprecedented. An examination of the effect of larval salt concentration on the transcriptome indicated that although several immune-related genes in *An. merus* were upregulated, the majority were downregulated in a dose-dependent manner [15]. Therefore, defensin-1 does not contribute to osmotolerance in this strain, but it does suggest that larval salt exposure would modulate the immune system, usually by reducing the expression of immune-related genes.

The effect of larval salt exposure on gut microbiota is largely unexamined. There are few studies on the species in general, and even fewer on the subject of the microbiota of this species. To the knowledge of the authors, there are two descriptions of microbiota in *An. merus* [61,62].

When examining α-diversity, variable larval salt concentrations resulted in a differential response in species richness and evenness. Species richness was not significantly affected by the treatments, but there were effects on species evenness. The most consistent differences were between low salt concentrations (0% and 6.25%) and the 50% treatment. Notably the diversity between larvae and adults were more similar in the high concentration than in the low concentrations. The lack of difference in species richness is not unexpected because the diversity was being compared in the same strain. Therefore, the intrinsic number of species does not differ, but the larval salt concentration does affect the distribution of those species.

There is a clear difference in β-diversity of the larvae reared in different salt concentrations and the adults that emerge from them. The marked reduction in β-diversity in adults is confirmed by the lower number of unique species in adults compared to larvae. This is due to the reduction in diversity associated with the renewal of the gut epithelia between pupae and adults [34]. However, examining β-diversity shows that not only is there a reduction in diversity from larvae to adult, but also that the diversity of the adults overlap, while that of the larvae does not (except for 50 and 100%). This suggests that the wide bacterial diversity in larvae may be associated with their surviving in the salty environment, and that the bacterial composition of the gut may play a role in this ability.

It is notable that the gut microbiome is not dominated by strong halophilic bacteria such as *Desulfovibrio*, *Halorubrum*, or *Natronomonas*. This does not mean that microbiota do not play a role in salt tolerance. The relevant halophiles may be concentrated in a different organ. The rectum and malphigian tubules play a more significant role in salt tolerance [14,63], and therefore, more halophilic bacteria may be concentrated there. However, *Klebsiella*, *Pseudomonas*, and *Ochrobacterum* are genera that have been found to be salt tolerant in plants [64]. It is therefore notable that when comparing the 50% seawater treatment to the 0% and 6.25% treatments, the high concentration had a significantly increased abundance of *Ochrobactrum*. By contrast, *Ochrobactrum* is not present in adults, nor was it represented in larvae reared in water without salt. This differed from the ubiquitous presence of *Klebsiella* or *Pseudomonas*. This suggests a role for *Ochrobactrum* in tolerating salt in *An. merus* larvae.

Despite the variability in gut microbiota due to salt treatment, there were 10 genera consistently represented in all treatments. *Bosea*, *Elizabethkingia*, *Enterobacter*, *Enterococcus*, *Klebsiella*, *Microbacterium*, *Rahnella*, *Sphingobacterium*, *Enterobacteriaceae*, and representatives of the *Allorhizobium*-*Neorhizobium*-*Pararhizobium*-*Rhizobium* group were present in all treatments. The latter represents *Agrobacterium* genera with a moderate salt tolerance [65]. Although not as highly overabundant in high salt treatment as *Ochrobacterum*, it is worth noting that they are not represented in adults, possibly representing another group of salt tolerant bacteria contributing to the osmotic tolerance of *An. merus*.

The ubiquitously representative genera, with the exception of *Bosea*, are represented in the guts of various other anopheline species (see, e.g., [66,67,68]). This adds to the body of evidence suggesting the existence of a core microbiome in the genus. *Elizabethkingia*, *Enterobacter*, *Enterococcus*, and *Klebsiella* have all been associated with various aspects of life history [69], potentially making them paratransgenesis candidates. It is also worth noting that *Rahnella* has been represented substantially in recent studies [35,62,70]. The role of this genera in life history is, however, poorly understood.

There is a range of bacterial genera associated with protection against *Plasmodium* species (see [31,32,71,72,73]). It can be expected that this is introduced to the mosquito gut during the aquatic life stage. This can be seen in this study where genera such as *Elizabethkingia*, *Pseudomonas*, and *Enterobacter* are present in the larvae. The variety of these *Plasmodium*-protective genera increases in adults. This suggests a trade-off between bacterial genera that may play a role in osmotolerance in the larvae, and genera that may play a role in *Plasmodium* modulation in the adult. It has been hypothesised that the change in diversity between larvae and adults may either be due to selection of particular species between pupation and emergence, or that some species are capable of colonising the newly emerged adult more rapidly [74]. The shift from genera that may assist surviving larval osmotic stress to genera associated with *Plasmodium* modulation suggests that the change is due to the change in mosquito biology rather than improved opportunistic colonisation.

There are various examples of increased bacterial diversity in the 50% seawater treatment. This is particularly notable in the relative abundance data, where adults from the 50% seawater treatment have significantly more genera represented. Notably, the number of *Plasmodium*-protective genera in these adults is far higher than in all the lower-salt counterparts. This may be a protective mechanism to compensate for the reduced immune function associated with breeding in highly saline waters [15].

In a previous study, the microbiota of the MAFUS strain used in this study were compared to those of laboratory *An. arabiensis* strains. It was demonstrated that this *An. merus* strain as well as an *Anopheles quadriannulatus* strain had significantly different bacterial diversity than the major vector *An. arabiensis*. Furthermore, it also had more *Plasmodium*-protective genera than the major vector [62]. This finding suggested a potential role for gut microbiota in vector competence. The findings of this study reinforced the findings of Singh et al., (2022), as similar patterns of microbiota were found. The combination of these findings along with the reduced immune gene expression (both in this study and in [15]) suggests that the osmotolerant capacity of *An. merus* may regulate immune function in this species and as such, potentially alter vector competence.

It is worth considering the real-life implications of this study. Although the strain is routinely reared in 17.5 g of salt, this trait is not fixed, and the tolerance can be reversed with prolonged breeding in fresh water (unpublished data). It is therefore not surprising that the highest salt concentration generally resulted in the greatest fitness advantage, with the notable exception of defensin expression. This does not necessarily mean that high salt concentrations in the field will result in greater fitness. The most likely scenario is that a change in the salinity between generations may result in decreased fitness unless a generational selection procedure is possible. What is important is that the larval saline exposure can alter the life history of this experience, and this makes it a consideration for surveillance where *An. merus* can be a potential vector. To fully understand the significance, particularly at the local level, the study would have to be repeated with wild local mosquitoes. The most practical suggestion arising from this study, therefore, is that the salinity of larval breeding sites where *An. merus* is found must be considered in surveillance exercises.

There are several limitations to this study. The most important is that this study was performed on a laboratory strain of *An. merus*. While some life history traits are comparable in wild and laboratory-reared strains, immunity is not one of them [75]. While the laboratory study may provide initial information of the microbial metagenomics, they will be an underrepresentation due to the reduced bacterial diversity in wild populations [76]. Additionally, this study used mRNA expression of a single antimicrobial peptide to examine the role of these peptides in osmotolerance and potentially immunity. To properly disentangle this effect, this must be examined at the protein level, as well as including more AMPs and other immune effectors, ideally from wild or F1 *An. merus*. Finally, it is important to note that although the study highlights statistical differences, which do not necessarily translate into functional effects in the field. This highlights the importance of incorporating the examination of larval environment, particularly where *An. merus* is present. The definitive way to determine whether our findings are of epidemiological significance is by confirmation from surveillance activities.

## 5. Conclusions

In conclusion, larval exposure to different salt concentrations alters the life history traits and gut microbiota of *An. merus*. High salt concentrations reduced the development time of the larvae but did not have a significant effect on adult longevity, fertility, or fecundity. Exposure to high salt concentrations increased deltamethrin tolerance and all larval salt exposure reduced the expression of *defensin-1*. Although there was a core microbiome, larval salt exposure altered the gut microbiota of both the larvae and the adults. The greater the difference in larval salt exposure, the greater the difference in comparative microbiota. Notably, the higher salt concentration had greater bacterial diversity as well as more bacterial genera associated with protection against *Plasmodium* parasites. As such, it is possible that the salinity of the breeding water of *An. merus* larvae may have the capacity to modulate the life history of this species in an epidemiologically significant manner. These findings suggest that the salinity of larval breeding water should be considered in surveillance efforts where *An. merus* is present

## Data Availability

All data associated with the manuscript are found in this publication.

## Supplementary Materials

The following supporting information can be downloaded at: https://www.mdpi.com/article/10.3390/insects13121165/s1, Figure S1: Bar plot of all specimens. Figure S2: rarefaction curves of larval specimens. Figure S3: rarefaction curves of adult specimens. Figure S4: Differential abundance in larvae. Table S1: Operational Taxonomic Unit (OTU) table of sequencing data; Table S2: Summary of metagenomic data as per the Mosquito Microbiome Consortium. Table S3: Primers used in this study. Table S4: Deltamethrin lethal times in the MAFUS strain.
