# Supplementary material for: Marked Effects of Larval Salt Exposure on the Life History and Gut Microbiota of the Malaria Vector Anopheles merus (Diptera: Culicidae)
Source: Insects. 2022 Dec 16;13(12):1165. doi: 10.3390/insects13121165 (PMC9787035; doi:10.3390/insects13121165)
Supplement: Supplementary file 1 [file insects-13-01165-s001.zip › Supplementary data/Table S2.docx]

**Metadata Summary**

Study Type: Laboratory

Number per sample: Pool of 3 individuals

Sample taxonomy: *Anopheles merus*

Developmental stage: 4^th^ instar larvae and adult

Sex: Female

Adult Age: 3-day post eclosion

Mating Status: Non-virgin

Blood feeding status: non-blood fed

Type of food provided: Beano/yeast mixture (larvae), 10% sucrose (adults)

Tissue processed: midgut

Sample phenotype: Plasmodium uninfected, low level insecticide tolerance

Biomolecule processed: DNA

Biomolecule isolation method: QIAGEN blood & tissue

Sequencing method: 16S rRNA amplicon

Sequencing platform: Illumina

Sequencing platform model: MiSeq (Illumina)

Sample storage preservative: None

Sample storage temperature: -20 °C

Sample storage duration: <1 month

Name and location of laboratory: Vector Control Reference Laboratory, Centre for Emerging Zoonotic and Parasitic Diseases, National Institute for Communicable Diseases, South Africa

Generation: In colony since 2012

Maintenance temperature: 25±2°C

Maintenance relative humidity: 80±5%

Light-dark cycle 12-h light; 12-h dark
