# Supplementary material for: Marked Effects of Larval Salt Exposure on the Life History and Gut Microbiota of the Malaria Vector Anopheles merus (Diptera: Culicidae)
Source: Insects. 2022 Dec 16;13(12):1165. doi: 10.3390/insects13121165 (PMC9787035; doi:10.3390/insects13121165)
Supplement: Supplementary file 1 [file insects-13-01165-s001.zip › Supplementary data/Table S3.docx]

**Supplementary Table 1:** **Primer sequences of the cDNA target, V3-V4 hypervariable regions and reference genes used in this study.**

| **Primer name** | **Primer sequence** |
| --- | --- |
| **Defensin-1 forward primer** | 5′ GGA CAA CTA GGA AGG ACA AAC A 3′ |
| **Defensin-1 reverse primer** | 5′’ ACG GTA GAG TCC TGA GGT AAA 3′ |
| **18S forward primer (Reference gene)** | 5′ TAC CTG GGC GTT CTA CTC 3′ |
| **18S reverse primer (Reference gene)** | 5′ CTT TGA GCA CTC TAA TTT GTT C 3′ |
| **26S forward primer (Reference gene)** | 5′ GAT AAG GCA ATC AAG AAG TTC G 3′ |
| **26S reverse primer (Reference gene)** | 5′ TAC GGA CAA CCT TCG AGT GG 3′ |
| **V3-V4 hypervariable region Forward Primer** | 5′ GTC TCG TGG GCT CGG AGA TGT GTA TAA GAG ACA GGA CTA CHV GGG TAT CTA ATC C 3′ |
| **V3-V4 hypervariable region Reverse Primer** | 5′ TCG TCG GCA GCG TCA GAT GTG TAT AAG AGA CAG CCT ACG GGN GGC WGC AG 3′ |
