# Supplementary material for: Marked Effects of Larval Salt Exposure on the Life History and Gut Microbiota of the Malaria Vector Anopheles merus (Diptera: Culicidae)
Source: Insects. 2022 Dec 16;13(12):1165. doi: 10.3390/insects13121165 (PMC9787035; doi:10.3390/insects13121165)
Supplement: Supplementary file 1 [file insects-13-01165-s001.zip › Supplementary data/Table S4.docx]

Table S4: Deltamethrin lethal times in the MAFUS strain. Error margins are given in brackets.

|  | **0%** | **12.5%** | **25%** | **50%** | **100%** |
| --- | --- | --- | --- | --- | --- |
| **LT50** | 14,33 (7.76; 29,42) | 18,29 (17,29; 44,88) | 16,79 (16,11; 26,78) | 24,69  (14,83; 43,09) | 29,46  (18,85; 49,09) |
| **LT99** | 17,14 (28,61; 131,71) | 25,48 (37,37; 203,64) | 29,41 (38,21; 71,08) | 24,78  (47,02; 134,38) | 61,71  (77,18, 244,53) |
