# Supplementary figures and images for: Marked Effects of Larval Salt Exposure on the Life History and Gut Microbiota of the Malaria Vector Anopheles merus (Diptera: Culicidae)
Source: Insects. 2022 Dec 16;13(12):1165. doi: 10.3390/insects13121165 (PMC9787035; doi:10.3390/insects13121165)

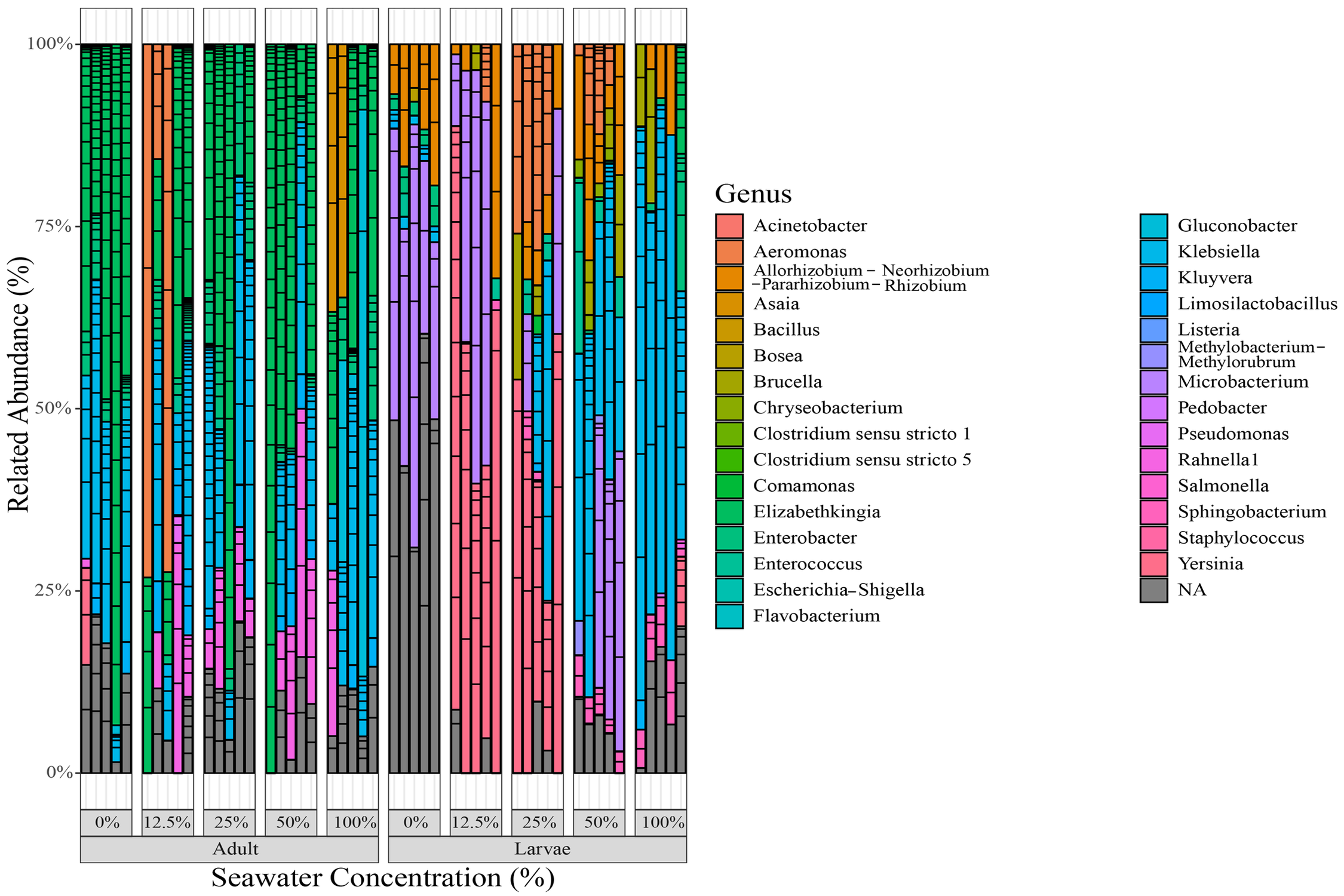

Supplement: Supplementary file 1 [file insects-13-01165-s001.zip › Supplementary data/Figure S1.tif]

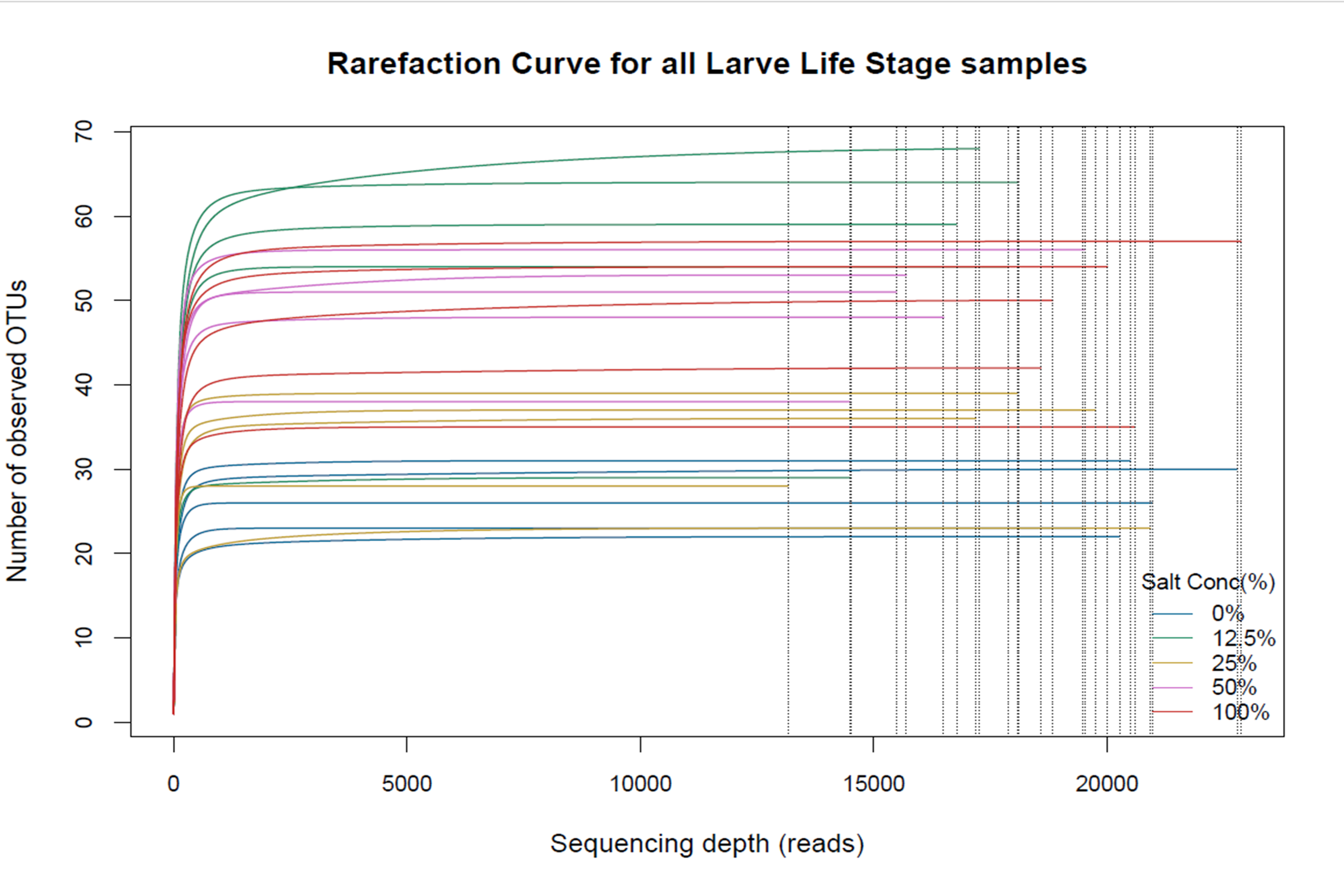

Supplement: Supplementary file 1 [file insects-13-01165-s001.zip › Supplementary data/Figure S2.tif]

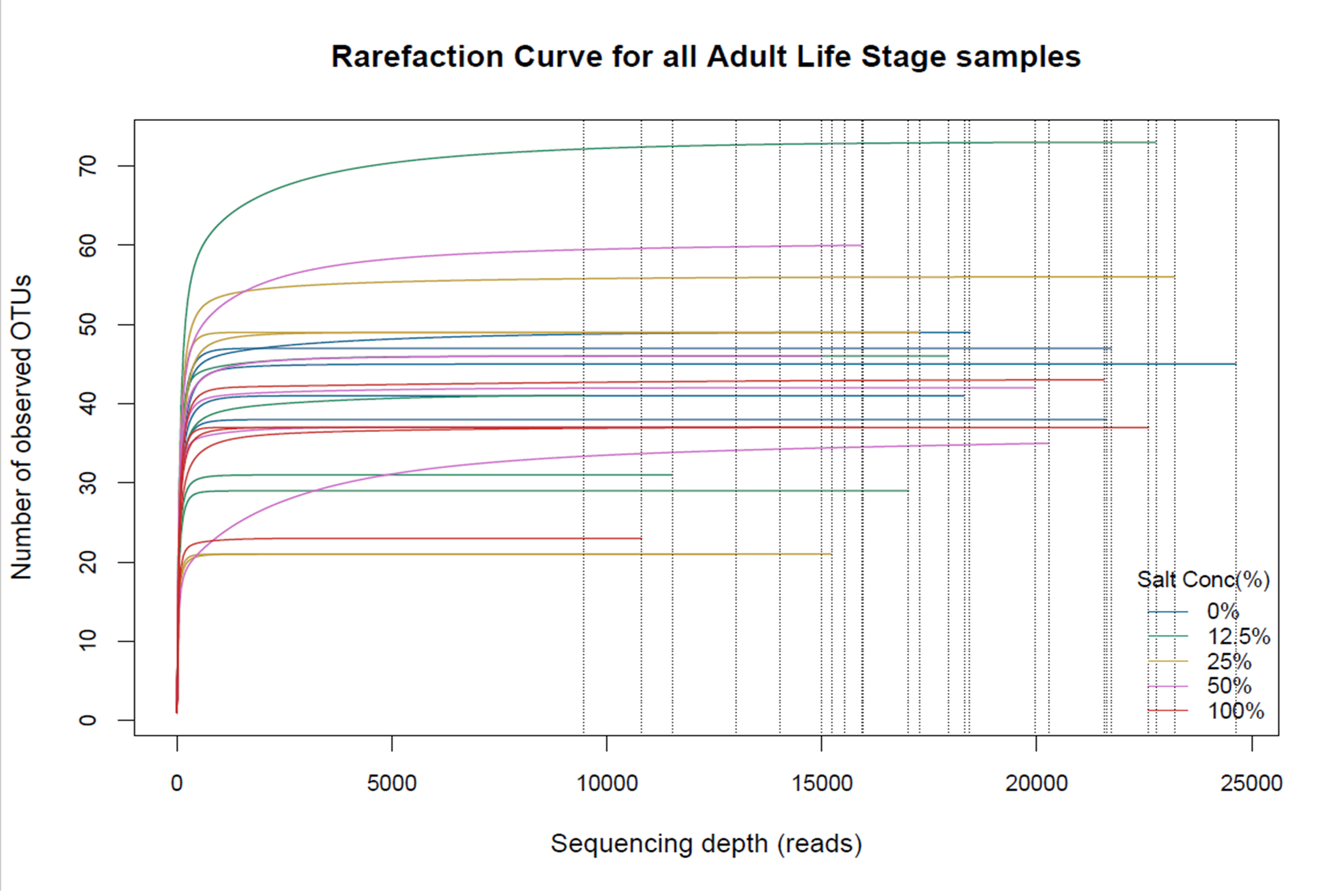

Supplement: Supplementary file 1 [file insects-13-01165-s001.zip › Supplementary data/Figure S3.tif]

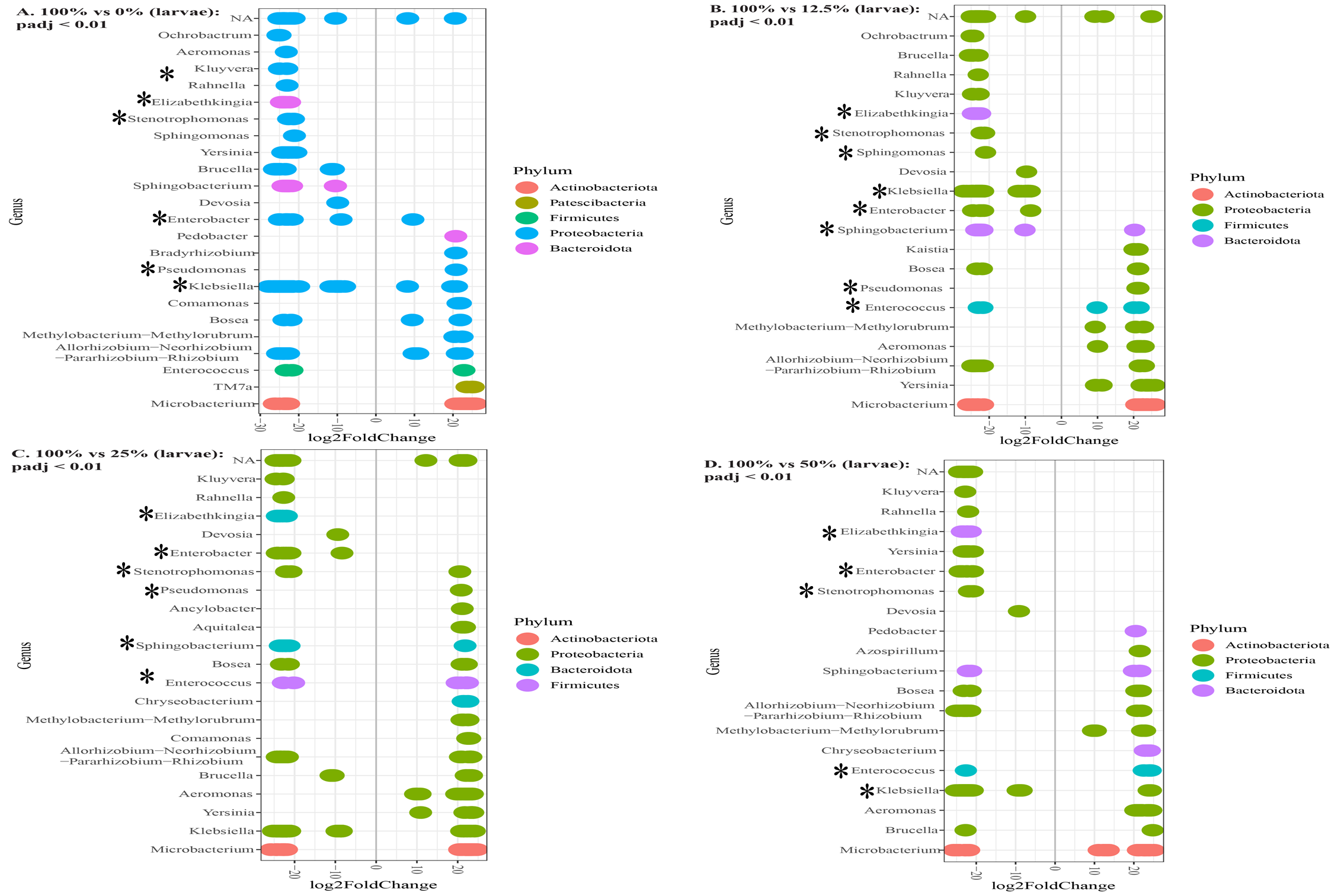

Supplement: Supplementary file 1 [file insects-13-01165-s001.zip › Supplementary data/Figure S4.tif]
